# Green Sonoextraction of Protein from Oleaginous Press Rapeseed Cake

**DOI:** 10.3390/molecules22010080

**Published:** 2017-01-04

**Authors:** Meryem Boukroufa, Anne-Gaëlle Sicaire, Frederic Fine, Colette Larré, Aude Le Goff, Véronique Solé Jamault, Njara Rakotomanomana, Farid Chemat

**Affiliations:** 1 GREEN Extraction Team, Avignon University, INRA, UMR408, F-84000 Avignon, France; meriem.boukroufa@alumni.univ-avignon.fr; 2 Terres Inovia, 33600 Pessac, France; ag.sicaire@terresinovia.fr (A.-G.S.); f.fine@terresinovia.fr (F.F.); 3 INRA, UR 1268 Biopolymeres Interactions Assemblages, 44316 Nantes CEDEX 3, France; colette.larre@inra.fr (C.L.); aude.le-goff@inra.fr (A.L.G.); veronique.sole@inra.fr (V.S.J.)

**Keywords:** green extraction, rapeseed, microwave, ultrasound, protein

## Abstract

In this study, extraction of soluble proteins from rapeseed cake using different conventional and innovative extraction processes in order to maximize the extraction yield has been investigated. Firstly, various extraction techniques including ultrasound, microwave, and percolation were tested to increase the protein recovery efficiency. Secondly, response surface methodology (RSM) using a central composite design (CCD) approach was applied to investigate the influence of process variables on ultrasound-assisted extraction (UAE). Statistical analysis revealed that the optimized conditions providing a protein yield of 4.24 g/100 g DM were an ultrasound power of 5.6 W·cm^−2^ and temperature of 45 °C. Quantitatively UAE followed by two stages of conventional extraction gave the best total protein yield of 9.81 g/100 g DM. Qualitatively, the protein efficiency ratio (PER) used as measure of the nutritive value (12S/2S ratio) which indicates protein quality in terms of S-containing essential amino acids, was similar to that of the conventional extraction method. Small amounts of protein aggregate were observed in the HPLC profile of the extract.

## 1. Introduction

Proteins, along with carbohydrates and lipids, are one of the three macronutrient families [1]. They consist of a sequence of 20 amino acids (AA) of which eight are considered as essential (i.e., not synthesized by humans) and that must be provided by food [2]. Two kinds of proteins can be distinguished: structural proteins (collagen, keratin) that participate in the constitution of tissues (bones, hair, skin) and proteins with biological activity which include enzymes, hormones, actin and myosin, transport proteins between cells and through cell membranes (membrane receptor, hemoglobin), proteins involved in host defense (immunoglobulin, fibrinogen), and reserve proteins [3]. Generally, this protein intake is of animal origin. However, according to a report from INSEE, meat consumption has been steadily decreasing since the 60s to reach 20% in 2014 against 26% when it was at its peak, with a greater orientation nowadays towards proteins of plant origin [4].

Rapeseed is primarily used for its oil. After that, the protein-rich meal, mainly composed of storage proteins such as albumin (napin) and globulin (cruciferin) [5] is generally used as a protein source in the livestock and aquaculture industries [6]. On the other hand, rapeseed has been indicated as a good alternative protein source for human nutrition due to its balanced amino acid profile and functional proprieties such as emulsifying, foaming and gelling abilities [3,7,8]. The use of rapeseed cake in human food is however complicated because of its content of antinutritional factors such as glucosinolates, phytic acid and phenolics [9]. Although some of these components can offer health benefits, other are not desirable. For example, phytate-protein complexes formed from phytic acid, are products which limit the human body’s digestibility [10].

Alkaline extraction with sodium hydroxide is the most widely used method to recover protein isolates from various matrices. This procedure is generally followed by a precipitation step by addition of a diluted acid. The solid liquid extraction step is performed by maceration with continuous stirring. The soluble fraction of proteins is obtained by modulating several operating parameters such as pH, temperature, and solid to liquid ratio. The mixture is then centrifuged in order to separate the meal residue from the extracted protein and the supernatant. Afterwards, the pH is adjusted to the isoelectric point in order to induce the precipitation of the soluble protein fraction [11]. However, proteins obtained with this procedure have poor solubility due to irreversible denaturation processes. A wide variety of protein extraction tools based on their physicochemical and structural characteristics, generally based on cell disruption, such as ultrasonic extraction [12], pulsed electric field extraction [13,14], high pulsed electric field [15], enzyme assisted extraction [16] or extraction by NAtural Deep Eutectic Solvents (NADES) [17,18] have been studied. On the other hand, these procedures must be optimized to minimize protein modification and denaturation.

**Figure 1:**
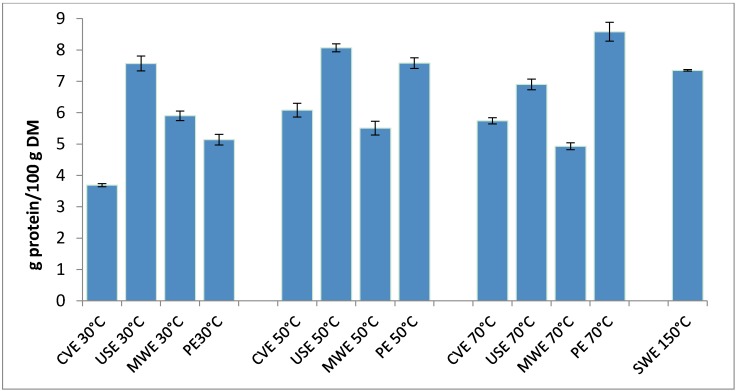
Protein yield profiles as a function of temperature.

In this work three extraction processes (microwave assisted extraction, ultrasound assisted extraction and percolation extraction) at three temperatures (30 °C, 50 °C, 70 °C) and Subcritical Water Extraction (SWE) will be compared to the conventional extraction process (Figure 1) in order to evaluate the effect of the extraction process on the recovery of protein from rapeseed cake. Then different protocols will be investigated to optimize the more valuable extraction process.

## 2. Results and Discussion 

### 2.1. Preliminary Study of Protein Extraction

The results of the parametric study of the extraction of the protein from rapeseed cake are shown in Figure 1. An optimal yield of 8.58 ± 0.05 g/100 g DM was obtained by using percolation in only 45 s of extraction vs. 8078.58 ± 0.05 g/100 g for ultrasound assisted extraction. Armenta et al. proposed a procedure using a hard cap espresso machine for the extraction of polycyclic aromatic hydrocarbons (PAHs) from contaminated soils and sediments and found comparable results by comparison with ultrasound-assisted extraction with an extraction time of 11 s, and a reduced amount of organic solvent to do the sample preparation [19]. 

A comparable yield was obtained with the ultrasonic assisted extraction for a temperature of 50 °C and an ultrasonic intensity of 5.7 W·cm^2^. The use of ultrasound assisted extraction enhanced the yield by 24.6% compared to the conventional extraction. These results are corroborated by those of Moulton and Wang [20] who reported that applying continuous high intensity ultrasound in combination with aqueous or alkaline extraction of protein from soybean meal resulted in the extraction of 23% more protein compared to conventional extraction. Dong et al. [12] also reported a 35.43% increase of protein extract yield from rapeseed by using an ultrasonic bath at a temperature of 35 °C.

On the other hand, subcritical water extraction allowed us to recover a protein yield of 7.35 g/100 g DM in only one extraction stage. In fact, water possesses many thermodynamic properties greatly influenced by temperature and pressure. Depending on the conditions, its physical state (solid, liquid or gas), thermal behaviour, density or viscosity can all be modified. By increasing the temperature and pressure over a point known as the critical point (defined as 221 bar and 374 °C), water can attain the supercritical state. Under subcritical conditions, corresponding to temperatures between 100 °C and the critical temperature and pressures between 1 bar and the critical pressure in order to avoid vaporization, water polarity decreases, which makes it a better solvent for the extraction of various organic bioactive compounds [21,22,23,24]. The more polar compounds are extracted at lower temperature and less polar compound are extracted at higher temperatures [25]. Watchararuji et al. [26] studied the application of subcritical water to the production of protein from raw and de-oiled soybean meal by subcritical water hydrolysis and showed that the optimal extraction conditions were 30 min of extraction time and a hydrolysis temperature of 210 °C and 200 °C for raw and de-oiled soybean meal, respectively. Wiboonsirikul et al. [27] obtained a better extraction yield of protein from soybean pulp by using an extraction temperature of 240 °C and an extraction time of 5 min. Both studies showed that the protein content in the extract increased with the increasing temperature treatment.

Microwave-assisted extraction of protein from rapeseed cake also is described to enhance the protein extraction yield. Choi et al. [28] observed that microwave treatment resulted in significantly higher concentrations of soluble protein from soybean meals than conventional treatment under optimum conditions. This intensification is due to the microwave thermal effect on the disruption of cell walls and tissues which help soluble protein diffusivity and enhance the extraction. However, as we can see in Figure 1, the protein yield decreases with increasing extraction temperature. This decrease can be explained by a possible denaturation of the extracted protein.

After this preliminary study, we decided to optimize the ultrasound-assisted extraction process because of its easy implemention in the food industries.

### 2.2. Statistical Analysis for UAE Extraction of Proteins According to the Experimental Design

The coded and decoded values of independent variables and protein yield obtained from the experimental design protocol are shown in Table 1.

**Figure 2:**
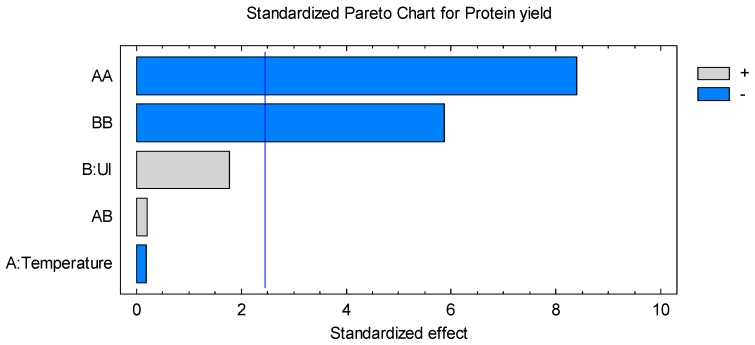
Standardized Pareto Chart for protein yield.

The standardized Pareto Chart diagram (Figure 2) illustrates the positive and negative effects of the factors on the protein yield, which is represented by horizontal bars. The vertical line indicates the significance of the effects at the 95% confidence level. From this figure, it can be deduced that there are two significant effects: the quadratic effect of the temperature (T) which is the most influential factor, followed closely by the quadratic effect of ultrasonic intensity (UI).

The experimental data obtained from the central composite design allowed us to build an empirical relationship linking the response studied to factors involved in the model. A positive value induces a promoting effect on the extraction, while a negative value indicated an antagonistic effect.

The second-order polynomial equation of the response surface obtained is as follows:

(1) $$Y_{protein}=0.91477+0.0908\;T+0.4303\;UI-0.0010\;T^{2}-0.0360\;UI^{2}$$

where *Y_protein_* is the protein yield (g protein/100 g DM), *UI* is the ultrasonic intensity (W·cm^−2^) and *T* is the temperature (°C). The coefficient of determination (R²) of the model indicates that the model as fitted explains 93.78% of the variability in protein yield, which indicates a close agreement between experimental and predicted values. The adjusted R-squared statistic, which is more suitable for comparing models with different numbers of independent variables, is 91.46%. 

**Figure 3:**
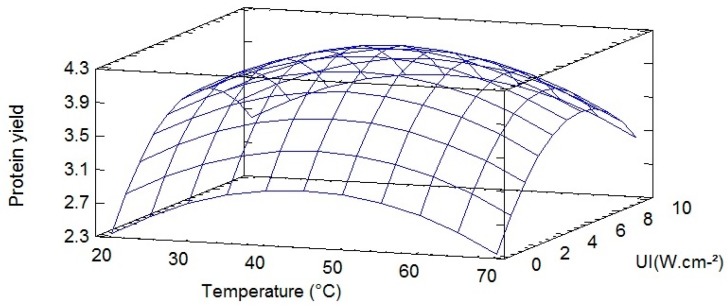
Response Surface Plots for protein yield depending on the temperature and the ultrasound power.

In order to visualize mathematical equation obtained by the software, a response surface was plotted and shown in Figure 3. By analyzing the surface plot obtained for protein yield as a function of temperature and ultrasonic intensity (Figure 3), we can see an increase of protein yield for a temperature range from 10 to 50 °C and an ultrasonic intensity range from 0.5 to 5 W·cm^−2^ after which the curve levels off. Indeed, the optimal settings for the maximization of the protein yield calculated by the software were a temperature of 45 °C and an ultrasonic intensity of 5.97 W·cm^−2^ (optimum calculated yield 4.24 g protein/100 g·DM).

### 2.3. Comparison between UAE and CE of Protein

In order to evaluate the impact of ultrasound-assisted extraction on protein yield, a comparison study was carried out between ultrasound and conventional extractions in optimized conditions obtained from the response surface methodology (T = 45 °C, UI = 5.6 W·cm^−2^) during one stage extraction.

**Figure 4:**
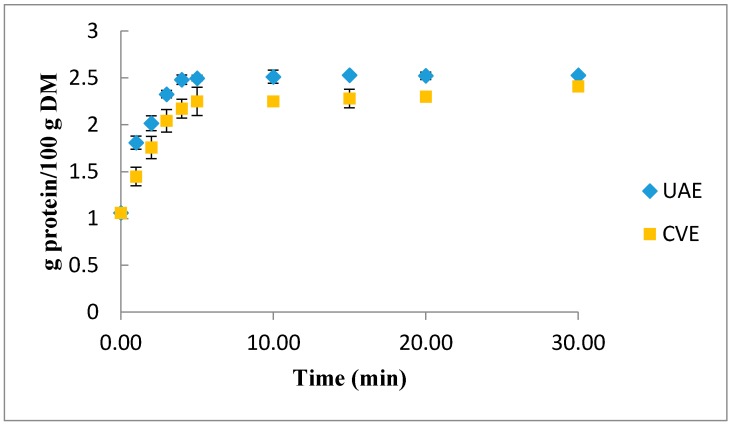
Comparison between conventional (CE) and ultrasound assisted extraction (UAE).

Extraction kinetics in the optimum conditions are shown in Figure 4. We can observe an improvement of UAE extraction. Indeed, we can see a small intensification generated by ultrasound-assisted extraction in shorter time than conventional extraction (5 min for ultrasound assisted extraction vs. 30 min for conventional extraction). This intensification is due to a mechanical, cavitation and thermal effect resulting from disruption of cell walls, practical size reduction and enhanced mass transfer across cell-membranes [29].

#### 2.3.1. Size Exclusion Chromatography

**Figure 5:**
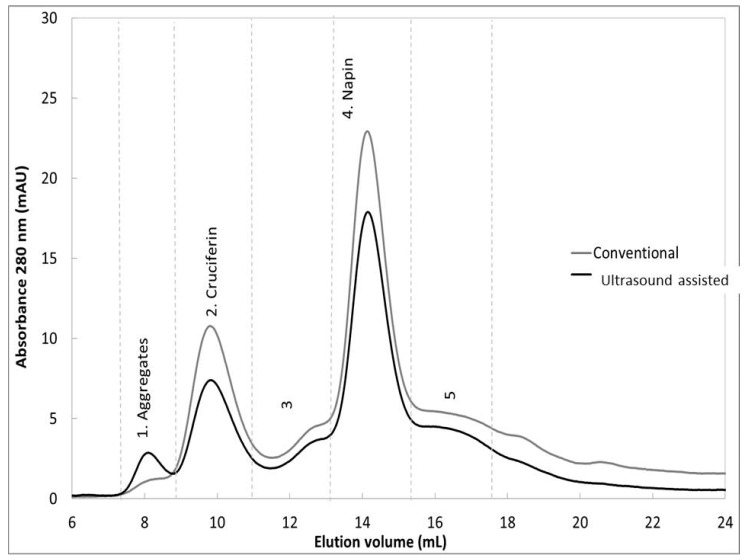
Seed storage protein content analysis by size exclusion chromatography (Superdex 75 column, Pharmacia, Broadway, CO, USA).

Depigmented extract obtained by the conventional and ultrasound assisted methods was injected at a flow rate of 0.5 mL/min in 50 mM Tris pH 8.5 750 mM NaCl buffer. Detection was performed at 280 nm (Figure 5).

From this figure we can see that qualitatively, the ultrasound assisted extraction doesn’t alter the protein extract. The 12S/2S ratio is similar to that of conventional extraction (Table 2). A small amount of protein aggregate is observed.

#### 2.3.2. Electrophoresis

**Figure 6:**
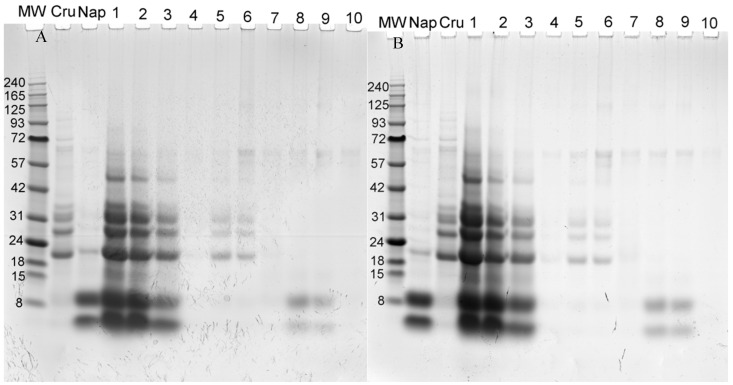
Electrophoresis pattern of fractions from conventional (**A**); or ultrasound-assisted (**B**) extraction under reducing conditions. MW molecular weight markers in kDa: (Nap) Napin standard, (Cru) Cruciferin standard, (1) extract; (2) filtered extract; (3) depigmented extract; (4) Superdex 75 peak 1 Aggregates; (5) and (6) Superdex 75 peak 2 Cruciferin; (7) Superdex 75 peak 3; (8) and (9) Superdex 75 peak 4 Napin; (10) Superdex 75 peak 5.

Proteins were separated on precast polyacrylamide 4%–12% Bis-Tris gels and stained with colloidal Coomassie blue (Figure 6). Each well contained the same volume of fractions.

This figure confirms the identity of compounds shown in Figure 5.

### 2.4. Multistage Cross-Current Simulation

**Figure 7:**
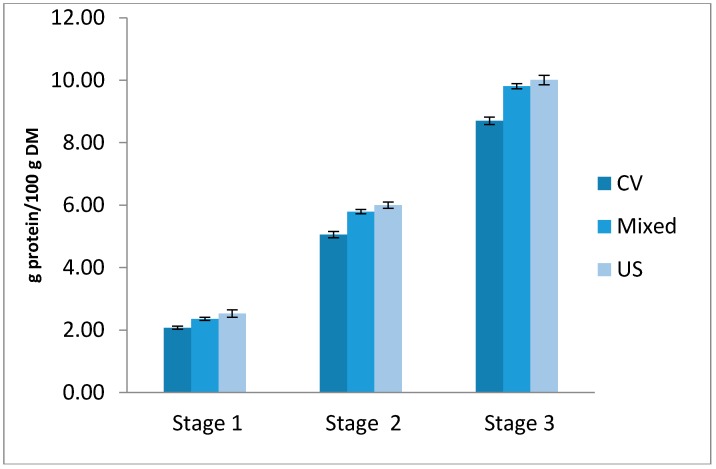
Cumulative extraction yields in multistage cross-current extraction: conventional (CV), under ultrasound (US) and mixed extraction conditions.

Figure 7 presents the extraction yields obtained for the three concepts. It can be concluded that a large amount of protein was extracted during the first extraction stage, but the US stage significantly extracted more than the conventional stage. It can be also observed that extraction of cake with only one stage of ultrasound assisted extraction followed by two stages of conventional extraction allowed us to obtain a similar protein yield as three ultrasound stages. These observations show that one ultrasonic stage is sufficient to disrupt the rapeseed cake structure and release the soluble proteins, so the total amount of solvent is likely to be reduced by two thirds.

## 3. Experimental Section

### 3.1. Plant Material

In this study, rapeseed cake was supplied by the Technical Institute of Oilseeds (Terres Inovia, Pessac, France). The initial moisture and protein content of the rapeseed cake was 5% and 33 g/100 g·DM respectively (protein content was evaluated by the Kjeldahl method [19] by Eurofins Analytics France (Nantes, France).

### 3.2. Chemicals

The solvents used were of analytical grade. Folin–Ciocalteu reagent was purchased from Sigma Aldrich (St. Louis, MO, USA). Modified Lowry Protein Assay Kit was purchased from Thermo Scientific (Rockford, IL, USA).

### 3.3. Protocol Treatment

#### 3.3.1. Conventional Extraction Procedure

The extraction was performed in a jacketed reactor in order to control the temperature of the medium by recirculating water using a heating cooling system. Rapeseed cake was sifted to 1 mm diameter and extracted with 10 volumes of deionized water for 30 min under agitation and pH of the medium was maintained at 7.4 during the experiment by using 1 M NaOH solution.

In order to determine the effect of temperature on the extraction process, three temperatures were studied: 30, 50, 70 °C. The extracts were then centrifuged at 5000 rpm for 10 min to obtain the water soluble fraction of proteins and the residue was similarly extracted twice again. All the experiments were performed on three extraction stages. Each extraction was performed three times.

**Figure 8:**
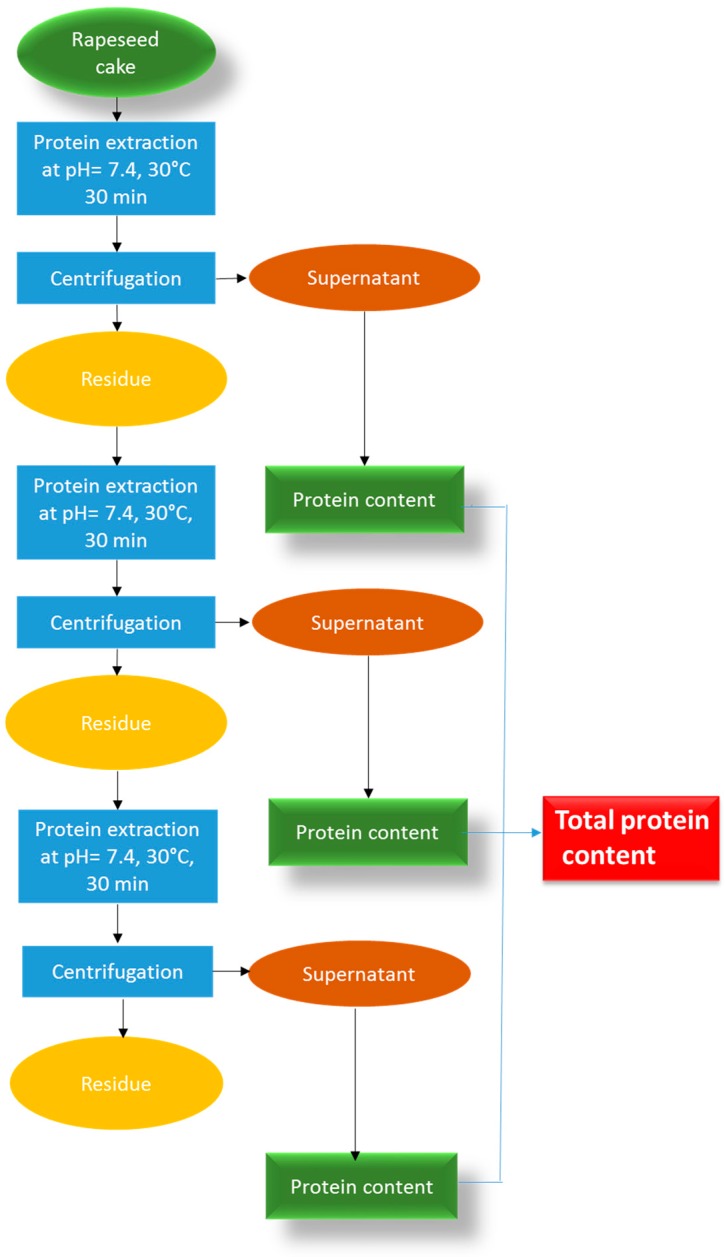
Conventional protocol extraction of proteins from rapeseed cake.

The experimental procedures used for the extraction of protein from rapeseed cake are shown in Figure 8.

#### 3.3.2. Ultrasound Assisted Extraction (UAE)

**Figure 9:**
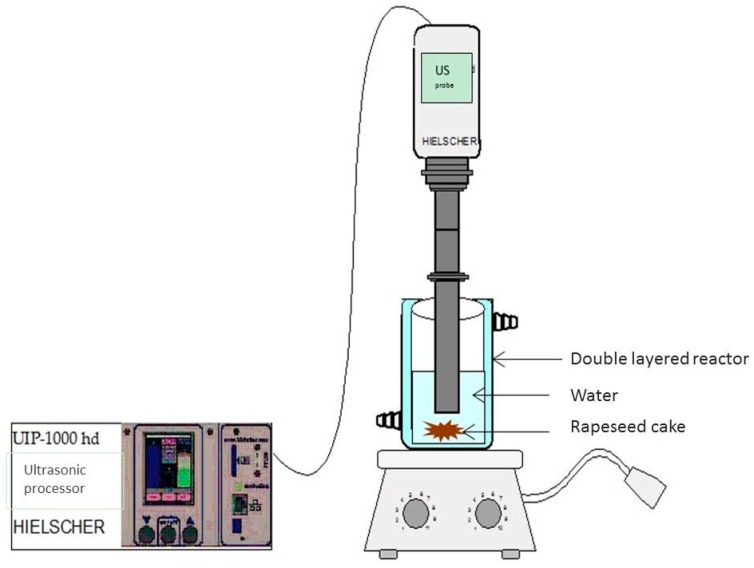
Ultrasound-Assisted Extraction of proteins from rapeseed cake.

The ultrasonic assisted extraction was performed with a Hielscher (Teltow, Germany) industrial ultrasonic processor UIP-1000 hd (frequency 20 KHZ, variable amplitude 25 µm, generator, 230 V), transducer IP65 grade with titanium horn and *O*-ring flange RFCA100 (stainless steel, diameter 100 mm) sealing to titanium sonotrode BS2d22 (length 125 mm, diameter 22 mm) (Figure 9).

For a rigorous comparison, the experiment was performed under the same operating conditions used for the conventional extraction. Each extraction was performed three times.

In order to take into account the power fraction converted to heat dissipated to the medium, calorimetric measurements were carried out to evaluate the real ultrasound power. The values of power were then calculated using Equation (2) [30]:

(2) $$P={m\;C}_{p}\;\frac{\mathrm{dT}}{\mathrm{dt}}$$

where m is the mass of solvent (g), C_p_ the heat capacity of the solvent at constant pressure (J·g^−1^·K^−1^), and dT/dt is the temperature variation according to time.

The applied ultrasonic power was then determined using the calculated power as given in the Equation (3) [30]:

(3) $$\mathrm{UI}=\frac{4P}{{\pi D}^{2}}$$

where UI is the ultrasonic intensity (W·cm^−2^), P the ultrasound power (W) calculated by Equation (2), and D is the diameter (cm) of the ultrasound horn.

#### 3.3.3. Microwave Assisted Extraction (MAE)

**Figure 10:**
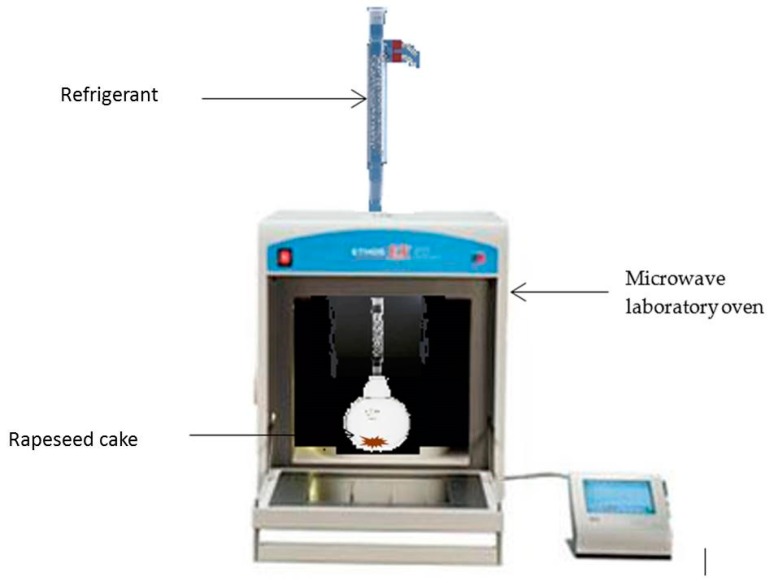
Microwave-Assisted Extraction of proteins from rapeseed cake.

Microwave assisted extraction has been performed in a Milestone EOS-G microwave laboratory oven (Milestone srl, Bergamo, Italy). It is a 2.45 GHz multimode microwave reactor with a maximum delivered power of 900 W in 10 W increments. (Figure 10). 

For a rigorous comparison, the experiment was performed under the same operating conditions used for the conventional extraction. Rapeseed cake was heated using a fixed power density of 1 W·g^−1^. Each extraction was performed three times.

#### 3.3.4. Subcritical Water Extraction (SWE)

**Figure 11:**
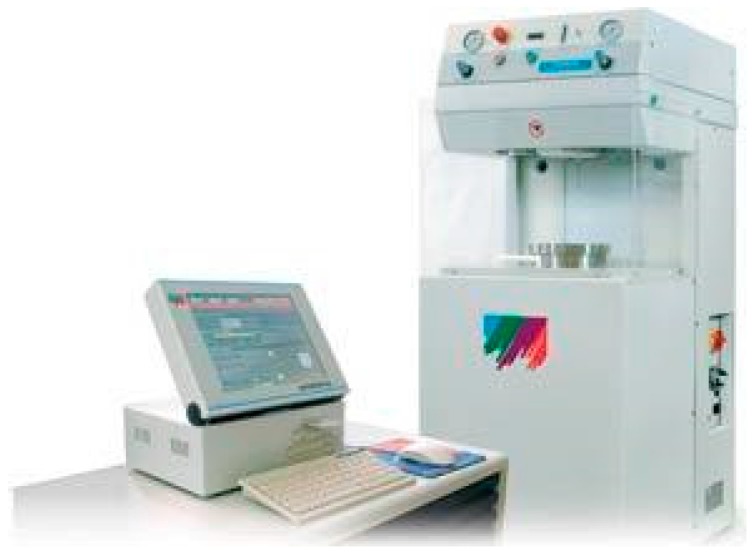
Subcritical water extraction of proteins from rapeseed cake.

The subcritical water extraction was performed using a 2.45 GHZ multimode microwave reactor with a maximum output power of 1200 W delivered in 10 W increments. The software allowed us to control temperature, power and time. A compressor connected to a nitrogen valve conditioned the initial pressure. The maximum pressure of the ultraclave was 200 bar and 220 °C (Figure 11).

The vessel containing 10 g of rapeseed cake with 100 mL of water was placed in the cavity. The initial working pressure of nitrogen was 10 bar. The working temperature of 150 °C is reached with a heating power of 1000 W. The temperature increase is achieved in 15 min, and then it is regulated for 30 min. Each extraction was performed three times.

#### 3.3.5. Percolation Extraction (PE)

**Figure 12:**
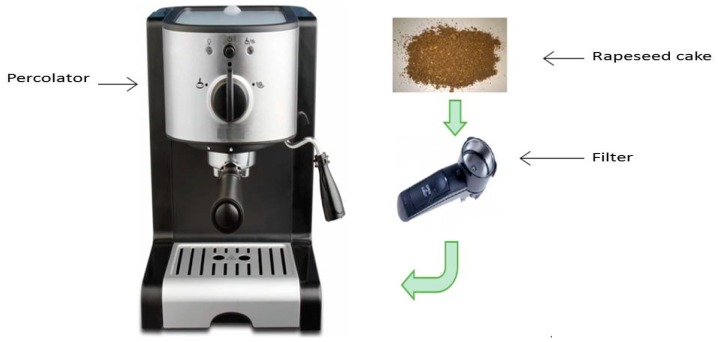
Percolation extraction of proteins from rapeseed cake.

A modified classical percolator (connected to a rheostat) was used (Figure 12). Rapeseed cake is extracted by the water at different temperatures for 15 s. Each extraction was performed three times.

### 3.4. Experimental Design

In order to determine the influence and performance of the ultrasound assisted extraction processing on protein recovery yield from rapeseed cake, response surface methodology (RSM) was employed. A Central Composite Design (CCD) was used to get maximal information about the process in a minimal number of experiments. The use of the multivariate study allows us to determine the interactions between variables and provides a complete exploration of the experimental study domain. The simple and quadratic effects and interactions of the operational parameters, namely temperature T (°C) (A); and ultrasound power expressed as the ultrasonic intensity UI (W·cm^−2^) (B) was studied using this design. The limit values of the ultrasonic intensity were chosen as function of limitations of ultrasonic apparatus (minimum and maximum power available in the device). Extraction temperatures were fixed between 20 °C and 80 °C. The latter was selected to check if there is any degradation or denaturation of the proteins. The coded levels and the natural values of the factors are shown in Table 1. A total of 12 different combinations including four replicates of the center point, designated by the coded value 0, four factorial points (2^2^) and four axial points (±α = ±1.41) were chosen in random order according to a CCD. The optimisation parameter was protein yield (Y_protein_) expressed as g protein/100 g dry matter (DM). The experimental designs used were constructed and the experimental results processed by using the Statgraphics Plus software (Version 5.1, Statistical Graphics Corporation, Rockville, MD, USA, 2000).

### 3.5. Multistage Cross-Current Simulation

**Figure 13:**
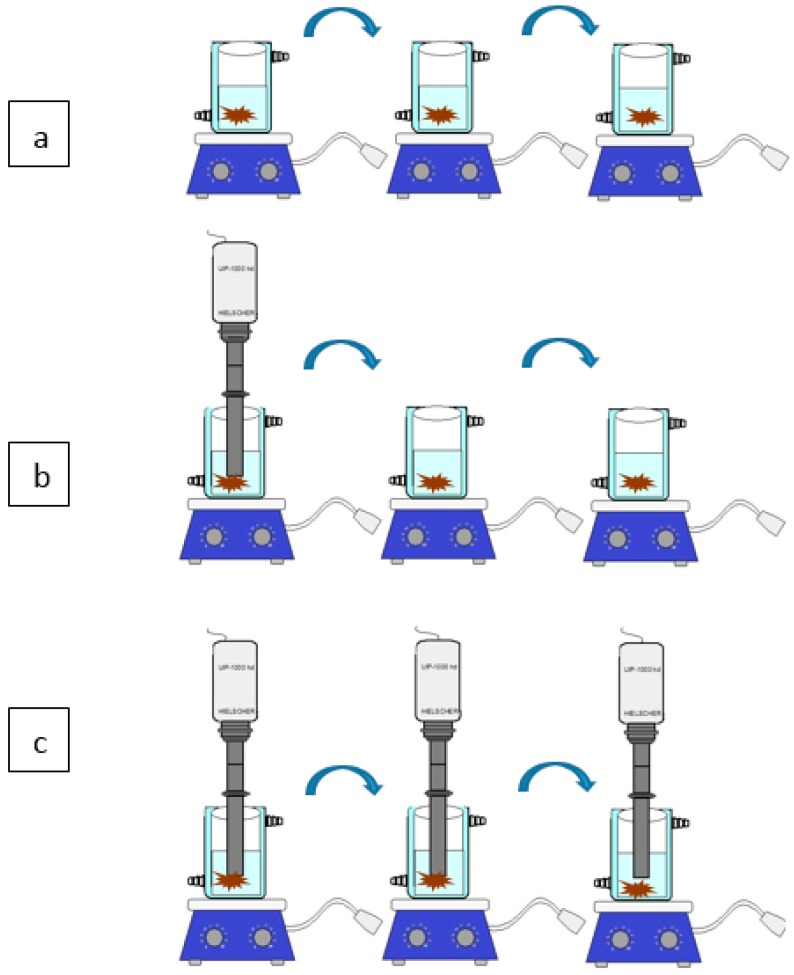
Multistage cross-current extraction; (**a**) multistage conventional maceration; (**b**) multistage extraction combination of (**a**,**c**); (**c**) multistage ultrasound assisted maceration.

Three concepts of multistage cross-current extraction (Figure 13) were implemented. Three stages of conventional extraction were compared with three stages of ultrasound-assisted extraction and a third mixed configuration with a first stage US followed by two conventional extractions. The experiments were performed in the same extraction conditions: Temperature = 45 °C, ratio = 1/10, and UI = 5.6 W·cm^−2^ for US. Each stage lasted 30 min.

### 3.6. Total Protein Yield (TPY)

The total protein yield was measured by the modified Lowry method with Bovine Serum Albumin (BSA) as standard. Each sample (200 µL) was added to 1 mL of modified Lowry reagent, mixed and incubated at room temperature for exactly 10 min. After that, 100 µL of 1 N Folin Ciocalteu reagent was added and mixed using a vortexer. The sample was then incubated at room temperature for 30 min. The absorbance was measured at 760 nm against a blank by using a UV/Vis spectrophotometer. Results were obtained by mg of BSA/100 g DM and were converted in g of proteins/100 g of DM by using the multiplication factor of 0.25. We got this factor from simultaneous Kjeldhal [31] and Lowry analysis of serial aliquots:

(4) $${\left[Proteins\right]}_{napin\;+\;cruciferin}\;=\;{\left[Proteins\right]}_{kdjeldhal}\;=\;{\left[Proteins\right]}_{Lowry}\;\times \;0.25$$

### 3.7. Gel Exclusion Analysis

The extract is filtered through 0.2 µm filter (Minisart RC, Sartorius, Bohemia, NY, USA). According to Bérot et al. [32], the filtrate (1 mL) is further depigmented by desalting chromatography on a Desalting column (5 mL, GE Healthcare, Velizy-Villacoublay, France). This fraction (500 μL) is separated using a Superdex 75 column (24 mL, Pharmacia) eluted with 50 mM Tris pH 8.5 750 mM NaCl buffer. Elution is carried out at 0.5 mL/min, detection is performed at 280 nm and the eluted fractions collected. The column is calibrated with cruciferin (12S globulin) and napin (2S albumin) standards injected in the same conditions. The area under each peak is integrated in order to quantify cruciferin and napin. The nature of each eluted peak is checked by SDS-PAGE electrophoresis under reducing conditions. Analyzes are performed in duplicates.

## 4. Conclusions

In this study, we conducted a technical study of different methods for extracting protein from rapeseed cake. The results obtained showed the ultrasound-assisted extraction offers a reduced extraction time and high extraction efficiency with a similar nutritive value (measured in Protein Efficient Ratio 12S/2S value) as conventional extraction. Multistage and mixed process optimization showed that one stage using ultrasound assisted extraction followed by two stages of conventional extraction were the best way to get the best yield of recovered protein from rapeseed cake.

## Figures and Tables

**Table 1:** Central composite design with their observed response.

Run	Coded Variables		Decoded Variables		Response
	A	B	Temperature (°C)	UI (W/cm^2^)	Y_Protein_ (g Protein/100 g DM)
1	0	0	45	5.5	**4.3**
2	+1	−1	70	2	**3.25**
3	0	0	45	5.5	**4.2**
4	0	+α	45	10.4	**3.43**
5	+1	+1	70	9	**3.45**
6	−1	−1	20	2	**3.22**
7	−1	+1	20	9	**3.34**
8	0	0	45	5.5	**4.25**
9	0	−α	45	0.5	**2.98**
10	+α	0	80.3	5.5	**2.74**
11	0	0	45	5.5	**4.2**
12	−α	0	9.6	5.5	**2.91**

**Table 2:** Seed storage protein composition in conventional or ultra-sound assisted extract.

Seed Storage Protein Peak Area	Conventional		Ultra-Sound Assisted	
	Mean	SD	Mean	SD
**Cruciferin (12S globulins)**	14.0	0.2	9.7	0.2
**Napin (2S albumins)**	28.3	0.4	22.7	1.0
**12S/2S ratio**	0.495	0.014	0.428	0.012
